# Supplementary material for: The interactome of intact mitochondria by cross-linking mass spectrometry provides evidence for coexisting respiratory supercomplexes
Source: Mol Cell Proteomics. 2017 Dec 8;17(2):216–32. doi: 10.1074/mcp.RA117.000470 (PMC5795388; doi:10.1074/mcp.RA117.000470)
Supplement: Supplemental Data [file supp_RA117.000470_133922_0_supp_23978_fzffwf.zip › spectra_annotation/mito_DR_spectra_annotation/4-2-10-1-10-1.pdf]

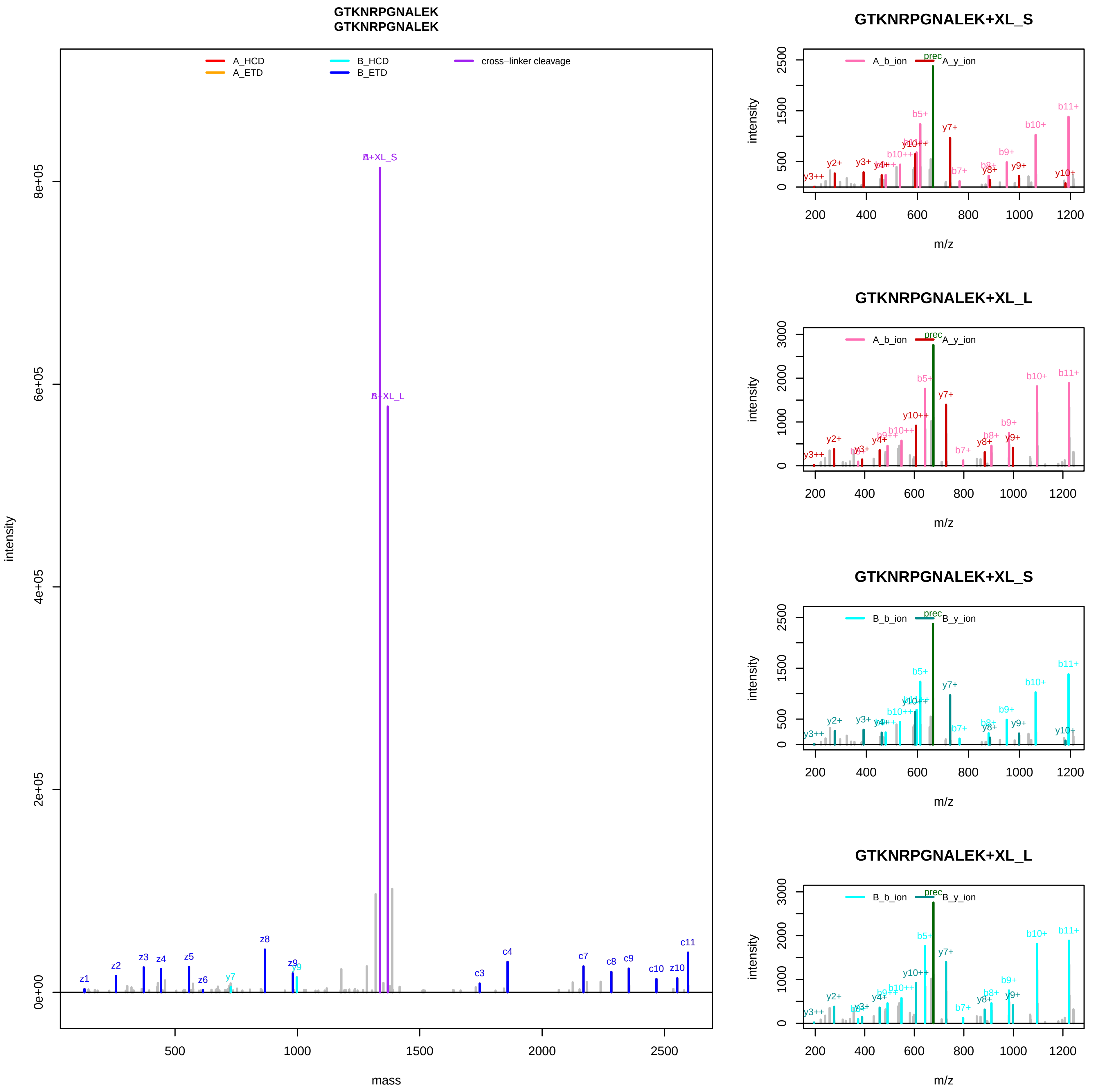

GTKNRPGNALEK
GTKNRPGNALEK
A_HCD
A_ETD
B_HCD
B_ETD
cross−linker cleavage
B+XL_S
B+XL_L
z1
z2
z3
z4
z5
z6
y7
z8
z9
y9
c3
c4
c7
c8
c9
c10
z10
c11
intensity
mass
0e+00
2e+05
4e+05
6e+05
8e+05
500
1000
1500
2000
2500
GTKNRPGNALEK+XL_S
A_b_ion
A_y_ion
prec
GTKNRPGNALEK+XL_L
A_b_ion
A_y_ion
prec
GTKNRPGNALEK+XL_S
B_b_ion
B_y_ion
prec
GTKNRPGNALEK+XL_L
B_b_ion
B_y_ion
prec
y3++
y2+
y3+
y4+
y7+
y8+
y9+
y10+
y10++
b5+
b7+
b8+
b9+
b10+
b11+
b9++
b10++
m/z
intensity
